# Humorous Physiology

**Published:** 1880-12

**Authors:** 


Humorous Physiology.—A report on the examination of girls in Boarding Schools, for the prizes offered by the National Health Society, was recently presented to the London School Board. The examination was attended by 215 girls from eleven schools at four centres, viz.: Medlum Street, Saffron Hill, Ben Jonson School, and Westmoreland Street. It is suggested as the reason why, out of 234 girls' schools, only eleven sent competitors, that physiology is taught as a specific subject in so very few girls' schools. And even in the representatives from these schools Mr. McWilliam, who held the examinations, noticed an abundance of faults.

Many of the children appear to have been utterly unable to understand the terms of the questions. "Mention any occupations which you consider to be injurious to health. giving reasons for your answer." This question, Mr. McWilliams says, especially appears to have puzzled them. One girl's comp'ete answer to this question is, "When you have a illness it makes your health bad, as well as having a disease." Another says, "Occupations which are injurious to health are carbolic acid gas, which is impure blood." Another complete answer is, "We ought to go in the country for a few weeks to take plenty of fresh air to make us healthy and strong every year." Another complete answer is, "Why, the heart, lungs, blood, which is very dangerous." The word "function" was also a great puzzle. Very many answered that the skin discharges a function called perspiration. One girl says, "The function of the heart is between the lungs." Another says, in answer to "What is the function of the heart?" "Thorax." Another girl in answer to the sixth question says, "The process of digestion is: We should never eat fat, because the food does not digest."

Another class of errors is that of exaggerated statements, one girl answering, "A stonemason's work is injurious, because when he is chipping he breathes in all the little chips, and then they are taken into the lungs." Another says, "A bootmaker's trade is very injurious, because the bootmakers always press the boots against the thorax, and therefore it presses the thorax in and it touches the heart, and if they do not die they are cripples for life." Several girls insist that every carpenter or mason should wear a pad over the mouth; and one girl says that if a sawyer does not wear spectacles he will be sure to lose his eyesight. Finally, one girl says that "all mechanical work is injurious to health." Another child says that "in impure air there is not any oxygen; it is all carbonic acid gas." Another says that if we do not wash ourselves "in one or two days all the perspiration will turn into sores."

One girl states that "when food is swallowed it passes through the windpipe and stops at the right side; some of it goes to make blood, and what is not wanted passes into the alimentary canal." Another girl from the same school says, "Venous blood is of a dark black color, and when it reaches the heart it is made by the heart a bright red color." Several girls from the same school repeat this last error. Another girl says, "The chyle flows up the middle of the backbone and reaches the heart, where it meets the oxygen and is purified." Another says, "The work of the heart is to repair the different organs in about half a minute." Another says, "We have an upper and a lower skin; the lower skin moves at its will, and the upper skin moves when we do."

In many of the papers errors in spelling are very numerous. One child says "The heart is a *comical* shaped bag." Another says "The upper skin is called *eppederby*, and the lower skin is called *derby*." Another says the organs of digestion are "Stomach, *utensils*, liver, spleen." Another speaks of the "*elementry cannal*." Another says that digestion is reducing our food into a "*plump*." Another says that in the heart "There is a fleshy *petition*, and it is divided into four parts, called the left *artilary*, the right *artilary*, etc." Of the simple word "*chew*" the inspector noted three distinct variations. One girl says, "First we put the food in our mouth, then it is *shewed;*

some people say our food is *shewed* 27 times." Another says, "The process of indigestion is that when we do not *eschew* our food enough it gives us indigestion." "The loss of teeth is a very serious matter, as we cannot *schew* our food enough." Another says, "First, before we can swallow any food, it *as* to be *jewed*, as *their* is a substance which helps to *jew* it, called saliva, and in that saliva *their* is a substance which is called ptyalin."

The errors, of which those mentioned above are samples, are confined for the most part to the papers of Standard IV. and in a less degree to those of Standard V. On the other hand, Mr. McWilliam says the papers of Standard VI. and Ex-Standard VI. girls are many of them very well written indeed.—*London Globe.*

